# Sleep and Epilepsy

**DOI:** 10.1155/2013/483248

**Published:** 2013-10-23

**Authors:** Andrea Romigi, E. Bonanni, M. Maestri

**Affiliations:** ^1^Sleep & Epilepsy Center, University of Rome “Tor Vergata”, General Hospital, Rome, Italy; ^2^Sleep Medicine Center IRCCS NEUROMED, Via Atinense 18, Pozzilli, Italy; ^3^Sleep Disorders Center, Neurological Clinic, University of Pisa, Pisa, Italy; ^4^Sleep and Epilepsy Center, Neurocenter of the Southern Switzerland, Civic Hospital of Lugano, Via Tesserete 46, Lugano, Switzerland

*“Of all the joys which are slowly abandoning me, sleep is one of the most precious, though one of the most common, too. A man who sleeps but little and poorly, propped on many a cushion, has ample time to meditate upon this particular delight. I grant that the most perfect repose is almost necessarily a complement to love, that profound rest which is reflected in two bodies. But what interests me here is the specific mystery of sleep partaken of for itself alone, the inevitable plunge risked each night by the naked man, solitary and unarmed, into an ocean where everything changes, the colors, the densities, and even the rhythm of breathing, where we meet the dead …”* [1].

Sleep is crucial to the health and well-being of all individuals, and is of particular relevance to patients with epilepsy. Sleep deprivation, daytime sleepiness, but also normal sleep *per se*, well-known potential triggers for seizures are themselves influenced by epilepsy in a sort of mutual effect. Comorbidities and pharmacological treatment are other commonly accepted major factors that influence this interplay. 

In clinical practice, patients with epilepsy are usually referred to sleep centers for either a specialistic evaluation of comorbid sleep disorders and a differential diagnosis between nocturnal seizures and parasomnias. Nonetheless, disrupted nocturnal sleep, excessive daytime sleepiness, and severe insomnia are common and frequently overlooked in these patients [2]. Sleep-related breathing disorders represent more than a simple comorbidity and should be considered a possible factor of pseudoresistance to antiepileptic treatment [3]. Sleep disruption may also worsen the neuropsychological outcomes in a population that has already an increased risk for cognitive and memory impairment. Diagnosing sleep alterations and sleep comorbidities in these patients is mandatory since these disorders are usually treatable or at least improvable by appropriate and individualized therapy. 

As stated in a recent review [4], although the intimate relationship between sleep and epilepsy has long been recognized, our understanding of the underlining mechanisms still is incomplete.

We selected for these supplement novel research articles or reviews about key and controversial issues in this field. P. Halasz provides a critical review regarding recent advances in the mechanisms underlying sleep and epilepsy networks, and their pathophysiological interplay. The authors draw modern conceptual updates and cast new light on the cognitive consequences of both of these phenomena. P. Halasz and coworkers reviewed the literature pertaining the potential interference of epileptiform discharges on slow-wave activity and NREM sleep microstructure dynamics and their plastic functions during sleep.

As previously hinted, sleep and antiepileptic drugs (AEDs) represent another puzzling variable. AEDs are a key factor of the mutual interactions between sleep and epilepsy, given their potential negative influences on the sleep-wake cycle and daytime vigilance [5]. V. Shvarts and S. Chung review the large body of literature investigating AEDs effects on sleep and face the emerging and intriguing fields of chronobiology and chronotherapy. While most of the conventional AEDs impair nocturnal sleep and daytime vigilance, novel AEDs may have minor or even positive impact on the sleep-wake cycle [6, 7]. F. S. Giorgi et al. present new clinical data on Lacosamide (a novel AED that acts selectively, enhancing slow inactivation of voltage-gated sodium channels) as an add-on therapy in a small cohort of drug-resistant epilepsy patients, finding no detrimental effects, on both subjective sleep quality and quantitative EEG parameters. 

In addition, it is well known that sleep modulates interictal abnormalities. NREM sleep and sleep deprivation facilitate epileptiform discharges, whereas REM sleep decreases not only the spiking rate but also the spatial distribution of EEG abnormalities [8]. Nonetheless, some controversies are still present in this field, and three different papers in this supplement deal with these issues. M. Ng and M. Pavlova carry out an extensive review of the literature on the frequency of seizures during REM sleep, confirming the protective role of EEG desynchronization against interictal abnormalities, focal and generalised seizures, and specific epileptic syndromes (i.e., Benign Epilepsy of Childhood with Rolandic Spikes). The clinical significance of sleep deprivation is still debated. A. D. Negrillo critically reviews the influence of sleep and sleep deprivation on seizures and interictal discharges, paying special attention to the major mechanisms highlighting this mutual interaction. On the other hand F. S. Giorgi et al. deal with a large number of peer-reviewed papers, looking for the answer to a more pragmatic question. What is the real role of EEG after sleep deprivation in the difficult diagnosis process of epilepsy? Despite the observed high methodological variability, heterogeneity of epilepsy syndromes, and lack of recent works, the review strongly supports the role and usefulness of sleep deprivation as a diagnostic tool for epileptologists.

In conclusion, these six papers represent novel steps in the challenging field of the relationship between sleep and epilepsy, and focus on some intriguing, unclear, and clinically significant issues.

*Andrea Romigi* *Andrea Romigi* *E. Bonanni* *E. Bonanni* *M. Maestri* *M. Maestri*
